# Post-reperfusion Syndrome and Outcome Variables after Orthotopic Liver Transplantation

**Published:** 2010-08-01

**Authors:** M. B. Khosravi, H. Sattari, S. Ghaffaripour, M. Lahssaee, H. Salahi, M. A. Sahmeddini, A. Bahador, S. Nikeghbalian, S. Parsa, S. Shokrizadeh, S. A. Malek-Hosseini

**Affiliations:** *Shiraz Organ Transplant Center, Namazi Hospital, Shiraz University of Medical Sciences, Shiraz, Iran*

**Keywords:** Postreperfusion syndrome, Severity, Outcomes, Transfusion, Orthotopic liver transplantation.

## Abstract

Background: Post-reperfusion syndrome (PRS) is an important complication during liver transplantation.

Objective: We studied the occurrence and severity of PRS in patients who underwent orthotopic liver transplantation (OLT) to investigate how PRS was correlated to clinical variables and outcomes.

Methods: We retrospectively recorded intra- and peri-operative data for 184 adult patients who received cadaveric OLT during a 3-year period from 2005 to 2008. Patients were divided into two groups according to the severity of PRS: Group 1 (mild or no PRS) comprised 152 patients; and group 2 (significant PRS) consisted of 32 patients.

Results: There were no significant differences in demographic and pre-operative data between groups. Group 2 had more total blood loss than group 1 (p=0.036), especially after reperfusion (p=0.023). Group 2 required more packed red cell transfusions (p=0.005), more fresh frozen plasma (p=0.003) and more platelets (p=0.043) than group 1. Fibrinolysis was more frequent in group 2 (p=0.004). Hospital stay in group 2 was significantly longer than in group 1 (p=0.034), but the frequencies of other outcomes including infection, retransplantation, dialysis, rejection and extended donor criteria did not differ significantly between groups.

Conclusions: Bleeding, blood transfusion and fibrinolysis occurred more often in the group of severe PRS after reperfusion. Although postoperative complications like rejection, infection and the dialysis rate were not significantly different in the two groups, hospital stay was more prolonged in the group with severe PRS.

## INTRODUCTION

Orthotropic liver transplantation (OLT) comprises three phases: 1) dissection to detach adhesions and mobilize the liver, 2) the anhepatic phase to remove the native liver and create vascular anastomoses with the transplanted organ, and 3) the neohepatic or reperfusion phase [1]. The reperfusion phase is the most critical time for anesthetists. Hemodynamic and metabolic events occur during reperfusion that are known as “post-reperfusion” or “post-revascularization syndrome” (PRS) [2]. The syndrome can also appear shortly after reperfusion of an ischemic tissue or organ. This complication should not be confused with ischemic reperfusion (IR) injury, which refers to local damage of a transplanted organ in response to prolonged ischemia [3].

PRS was first reported by Starzl, *et al*. and was described by Aggarwal, *et al*., in 1987 [2] as cardiovascular collapse after reperfusion of the transplanted liver. They defined a syndrome of severe cardiovascular dysfunction, bradycardia, decreased mean arterial pressure (MAP) and systemic vascular resistance, with a simultaneous increase in pulmonary filling pressures. PRS was more recently defined as a decrease in MAP (<70% of the reperfused value) for a minimum of 1 min within 5 min of reperfusion [2,4]. However, Hilmi, *et al*., pointed out that some degree of hemodynamic instability was seen in all patients. Recently, PRS has been classified according to its duration and severity [5]. The etiology of PRS is not clearly understood, but the syndrome has been attributed to different factors including metabolic acidosis, hyperkalemia, hypothermia, hypocalcemia and the release of vasoactive substances [2,6]. The incidence of PRS is about 8%–30% in patients who receive OLT [4,7,8].

In this retrospective study we investigated PRS in patients who received OLT and looked for relationships between the severity of this syndrome and potentially problematic post-reperfusion changes, the amounts of blood loss and transfusion, short-term outcomes, and post-operative complications.

## METHODS

For conducting this retrospective study, we obtained institutional approval to review the anesthesia records and peri-operative data of 184 consecutive patients >14 years old who underwent cadaveric donor OLT by the piggyback hepatectomy technique during a three-year period from 2005 to 2008. Two groups of patients were defined according to the decrease in MAP or heart rate after reperfusion in relation to the baseline value recorded 10 min before portal vein declamping and reperfusion. Group 1 comprised 152 patients with mild PRS manifested as a decrease in MAP or heart rate less than 30% of the anhepatic level that lasted ≤5 min. This group also included patients who responded to an intravenous epinephrine bolus (≤100 μg) or intravenous calcium chloride (1 g), and did not need the continuous infusion of the vasopressor or inotrope. Group 2 consisted of 32 patients with severe PRS manifested as decrease in MAP >30% of the anhepatic value and severe bradyarrhythmia requiring continued vasopressor infusion after reperfusion and prolonged fibrinolysis (>30 min) [5].

We recorded demographic characteristics and preoperative laboratory values. Intra-operative hemodynamic changes, transfusion need before and after reperfusion, vasopressor usage, incidence of fibrinolysis (assessed by thromboelastogram), short-term postoperative outcomes and complications were recorded and compared between the two groups. The extended donor criteria (EDC) for liver allograft quality were: age >65 years, serum sodium level >155 mEq/L, donor liver macrosteatosis ≥30% on biopsy, warm ischemic time >90 min and cold ischemic time >16 h.

*Student’s t *test for independent samples and Mann-Whitney U test were used for comparison of continuous data. For categorical variables, we used χ^2^ or Fisher exact test when appropriate. The data are presented as the mean±SD or the median. All analyses were done with SPSS v. 15. P value <0.05 was considered statistically significant.

## RESULTS

Some degree of PRS was observed in all 184 patients. Among them, 152 (82.6%) patients were classified as group 1 (mild PRS), and 32 (17.4%) patients were classified as group 2 (severe PRS). We found no significant differences between groups in demographic or pre-operative data (Table 1).

**Table 1:** Characteristics of patients before orthotopic liver transplantation

**Characteristics**	**Group 1 (** ***n*** **=152)**	**Group 2 (** ***n=*** **32)**	***P *** **value**
Age (year)	34.44±13.23	36.88±13.59	.348
Male	67.8%	62.5%	.352
Female	32.2%	37.5%	.352
Weight (kg)	64.12 ±14.49	63.62± 15.72	.861
Serum creatinine (mg/dL)	.98± .47	.84± .50	.128
Platelet count (x1000)	98.48± 106.10	85.91± 63.23	.519
INR	2.77± 1.94	2.34 ±1.37	.843
Hematocrit	34.25± 4.32	34.13± 3.98	.608
MELD	21.75± 5.50	21.56± 8.25	.903
AIH	17.8%	18.8%	.887
HBV	24.3%	34.3%	.887
HCV	7.1%	6%	.887
PSC	17.1%	15.6%	.887
Wilson disease	11.2%	9.4%	.887
Cryptogenic	21.7%	15.6%	.887

INR: international normalized ratio, MELD: model for end-stage liver disease, psc: primary sclorosing cirrhosis, AIH: autoimmune hepatitis, HBV: hepatitis B cirrhosis, HCV: hepatitis C cirrhosis.

Comparison of the intra-operative variables revealed that group 2 patients had more total blood loss than those in group 1 (p=0.036)—a difference that reflected the greater post-reperfusion blood loss in group 2 (p=0.023). Before reperfusion, the amount of blood loss did not differ significantly between groups. Group 2 patients used more packed red cells (PRBC) than group 1 patients (p=0.005)—a difference that was especially marked after reperfusion (p=0.003). Group 2 patients used higher amounts of fresh frozen plasma (FFP) (p=0.003) and platelet (p=0.043). The incidence of fibrinolysis in group 2 was more than in group 1 (p=0.004). The mean cold ischemic time (CIT), warm ischemic time (WIT), the amount of sodium bicarbonate (NaHCO_3_) used, and serum potassium concentration after reperfusion did not differ significantly between groups (Table 2). Donor characteristics including mean age, serum sodium concentration, and duration of stay in the intensive care unit also showed no significant differences between groups (Table 3). The post-operative data showed that hospital stay was however significantly longer in group 2 patients (p=0.034). Post-operative outcomes including infection, retransplantation, dialysis, rejection and serum creatinine, and the EDC, did not differ significantly between groups (Table 4).

**Table 2:** Intra-operative data during orthotopic liver transplantation

**Intra-operative Data **	**Group 1**	**Group 2**	***P *** **value**
CIT (hours)	10.29±6.73	8.93± 4.08	.274
WIT (minutes)	63.28± 13.67	67.97± 11.25	.072
PRBC before reperfusion (units)	1.87± 1.60	2.78± 2.69	.074
PRBC after reperfusion (units)	1.82± 1.62	4.72± 4.98	.003*
Total PRBC (units)	3.66± 2.47	. 7.50± 7.18	.005*
FFP (units)	1.11± 2.33	4.00± 5.15	.003*
PLTS (units)	1.03± 2.46	3.15± 5.58	.043*
NaHCO3 ( mL)	218.95± 122.19	247.69± 175.13	.095
K after reperfusion	4.08± .97	4.24± 1.04	.407
Fibrinolysis	6.6%	25%	.004*
Blood loss before reperfusion ( mL)	1000 (100-6500) †	1300 (100-8000) †	.187
Blood loss after reperfusion ( mL)	1000 (100-10000) †	1450 (150-24500) †	.023*

CIT: cold ischemic time, WIT: warm ischemic time, PRBC: packed red blood cells, FFP: fresh frozen plasma, PLTS: platelets, NaHCO_3_: sodium bicarbonate, K: serum potassium.

* significant at *P*=.05.

† : Median and range of blood loss.

**Table 3:** Donor characteristics

**Donor characteristics**	**Group 1**	**Group 2**	***P *** **value**
Age (year)	30.19± 14.26	33.13± 14.25	.292
Na	147.46± 10.53	149.92± 13.29	.291
ICU stay (day)	3.01± 1.94	2.84± 1.96	.659
Male	81.5%	18.5%	.552
Female	85%	15%	.552

ICU: intensive care unit, Na: serum sodium.

**Table 4:** Post-operative data for patients who underwent orthotopic liver transplantation

**Postoperative data **	**Group 1**	**Group 2**	***P *** **value**
Hospital stay (day)	14.93 ± 6.98	19.19 ±10.27	.034*
Infection	9.9%	3.1%	.313
Retransplantation	0%	3.1%	.174
Dialysis 1st month	5.3%	9.4%	.409
Rejection 1st month	32.9%	25%	.530
Cr first postoperative day	.85 ±.46	.85 ±.48	.999
EDC Graft	26.8%	37%	.351

EDC: extended donor criteria, Cr: serum creatinine.

* significant at *P*=.05.

## DISCUSSION

PRS was first defined in liver transplantation by Aggarwal, *et al*., in 1987 [2]. It is a widely reported event that can occur after the reperfusion of an ischemic organ. In different studies, many contributing factors have been reported. Hilmi, *et al.* [5], found that the age of the recipients differed significantly—patients with significant PRS were older. The age difference between our two groups was not significant, although our patients were younger. Comparison of the demographic and preoperative data between the two groups revealed no significant differences. Studies by Nanshima, *et al.* [9] and Ayanoglu, *et al.* [10] also found no significant differences between pre-operative characteristics. Nanshima and colleagues also found that WIT in the PRS group was longer which was not significant. Hilmi, *et al*., reported shorter WIT in the significant PRS group compared to mild PRS (p=0.010) [5].

Ayanoglu and colleagues found that longer anhepatic time was associated with decreased PRS occurrence. Longer WIT, especially >90 min, can cause severe ischemic insult and worsen the extent of injury during reperfusion [11,12]. However, in our study WIT was optimal and well within the acceptable range. Intra-operative data showed that WIT in group 2 patients (significant PRS) was longer than in group 1 although this difference was not significant (Table 2).

CIT was shorter in our group 2 patients, although the difference was not significant. Most graft insults occur during reperfusion [13,14], although the initial insult begins during CIT due to mitochondrial dysfunction and cellular membrane damage [15]. Oxidative stress at reperfusion time leads to activation of Kupffer cells and microvascular dysfunction (no-reflow), and to neutrophil activation [16]. However, ischemic reperfusion (I/R) injury may result in failure or primary nonfunction of the transplanted organ. The I/R injury may or may not be the cause of hemodynamic changes immediately after reperfusion (PRS), and the relationship between the I/R injury and PRS has yet to be clearly characterized [17].

In our study blood loss and blood product usage were significantly more in group 2, and in both groups blood loss and transfusion were greater after reperfusion than before it. The greater blood loss and transfusion need in group 2 were associated with more severe and frequent fibrinolysis in this group (Table 2). Fibrinolysis can increase after reperfusion due to increasing tissue-type plasminogen activator (tPA) activity [18]. Blood transfusion can worsen the outcome in patients and in the transplanted organ [19]. The lower incidence of rejection in the first post-operative month in group 2 was notable, and may have been due to the higher transfusion volume and greater immunosuppressant effects of blood transfusion in this group [20].

The amount of sodium bicarbonate used and serum potassium level after reperfusion did not differ significantly between groups. This finding was similar to the report of Nanshima, *et al*. [9]. Aggarwal and colleagues also found no significant differences between groups in terms of serum potassium level, incidence of acidosis, serum calcium concentration, core temperature, or arterial blood gas tensions except for a decrease in systemic vascular resistance [4].

Our two groups did not differ in terms of the frequency of post-reperfusion complications except for the significantly longer hospital stay in patients with severe PRS. This finding is consistent with earlier results reported by Hilmi, *et al.* [5], and Nanshima, *et al.* [9].

Compliance of the grafts that were used with EDC did not differ significantly between our two groups; the use of these grafts was unrelated to the severity of PRS. Nanshima and colleagues found that donor age >50 years was associated with the occurrence of PRS, but did not affect patient or graft outcome. However, PRS may affect patient and graft outcomes, hence, the need to prevent its associated adverse hemodynamic and metabolic effects and thus improve outcomes. Different approaches are available to reduce reperfusion injuries. Examples are administration of vasodilators such as inhaled nitric oxide, prostaglandins, free radical scavengers, ischemic preconditioning, and use of therapeutic substances such as N-acetylcystine and methylen blue.

We conclude that during OLT, blood loss, transfusion and fibrinolysis were higher in the group with severe PRS after reperfusion of the transplanted liver. Although post-operative complications like rejection, infection and the dialysis rate were not significantly different in the two groups, hospital stay was more prolonged in the group with severe PRS.
